# Additive and Non-Additive Effects on the Control of Key Agronomic Traits in Popcorn Lines under Contrasting Phosphorus Conditions

**DOI:** 10.3390/plants11172216

**Published:** 2022-08-26

**Authors:** Talles de Oliveira Santos, Fábio Tomaz de Oliveira, Antônio Teixeira do Amaral Junior, Janeo Eustáquio de Almeida Filho, Rosimeire Barboza Bispo, Marta Simone Mendonça de Freitas, José Francisco Teixeira do Amaral, Samuel Henrique Kamphorst, Valter Jário de Lima, Flávia Nicácio Viana, Guilherme Ferreira Pena, Pedro Henrique Araújo Diniz Santos, Wallace de Paula Bernado, Messias Gonzaga Pereira, Jurandi Gonçalves de Oliveira, Ricardo Enrique Bressan-Smith, Roberto dos Santos Trindade

**Affiliations:** 1 Plant Genetic Breeding Laboratory, Center for Agricultural Sciences and Technologies (CCTA), Universidade Estadual do Norte Fluminense Darcy Ribeiro (UENF), Campos dos Goytacazes 28013-602, RJ, Brazil; 2 Bayer, Estrada da Invernadinha, 2000, Coxilha 99145-000, RS, Brazil; 3 Plant Science Laboratory, Center for Agricultural Sciences and Technologies (CCTA), Universidade Estadual do Norte Fluminense Darcy Ribeiro (UENF), Campos dos Goytacazes 28013-602, RJ, Brazil; 4 Department of Rural Engineering, Center for Agronomic Sciences and Engineering, Universidade Federal do Espírito Santo (UFES), Alegre 29500-000, ES, Brazil; 5 National Research Center for Maize and Sorghum, Brazilian Agricultural Research Corporation, MG-424 Highway, Km 45, Sete Lagoas 35701-970, MG, Brazil

**Keywords:** abiotic stress, genetic control, Griffing diallel analysis, *Zea mays* var. *everta*

## Abstract

Phosphorus is a non-renewable natural resource that will run out of reserves in the upcoming decades, making it essential to understanding the inheritance of nutrient use efficiency for selecting superior genotypes. This study investigated the additive and non-additive effects of commercially relevant traits for the popcorn crop (grain yield—GY, popping expansion—PE, and expanded popcorn volume per hectare—PV) in different conditions of phosphorus (P) availability in two locations in Rio de Janeiro State, Brazil. Six S_7_ lines previously selected for P use—L59, L70, and P7, efficient and responsive; and L54, L75, and L80, inefficient and non-responsive—were used as testers in crosses with 15 progenies from the fifth cycle of intrapopulation recurrent selection of UENF-14, with adaptation to the North and Northwest regions of Rio de Janeiro State. Using the Griffing diallel analysis, P use efficiency was predominantly additive in the expression of PE, and non-additive effects were prominent for GY and PV. For obtaining genotypes that are efficient for phosphorus use, it is recommended that heterosis with parents that provide additive gene accumulation for PE be explored.

## 1. Introduction

Phosphorus (P) is the second most consumed nutrient in agriculture, surpassed only by nitrogen, and is limiting for agricultural productivity worldwide [1]. Despite playing a crucial role in crop productivity, its soil reserves (organic and inorganic forms) have limited supply to the plants because of its fixation and formation of complexes with other soil nutrients [2,3].

Maize (*Zea mays*) is one of the most widely cultivated species of commercial interest, both as a staple food and for industrial use, in tropical and temperate climatic soils in the world. Under cultivation, especially in acidic and alkaline soils, large amounts of P fertilizer are applied to maize fields to maximize yields. The consequence is high amounts of high-cost phosphate fertilizers being applied to P-deficient soils to achieve maximum yields to guarantee food security for the growing world population, which will hit 10 billion inhabitants by 2050 [4]. Therefore, to meet this demand, the global production of phosphate fertilizers will require a significant increase in phosphate extraction in the next decades. This is, however, a non-renewable resource that is likely to become scarcer as its use becomes more frequent [5].

Accordingly, promoting phosphorus use efficiency in crop plants is key to sustainable agricultural development; this is especially important in tropical and subtropical regions, where there is greater loss of the nutrient due to high temperatures and heavy rainfall, together with the fixation of the nutrient by iron and aluminum oxides in the soil, resulting in the loss of about 70–80% of the nutrient applied to crops [6,7,8].

Phosphorus deficiency in soil leads to physiological and biochemical disturbances in plants. As a result, they show a reduction in leaf area, height, dry matter, and major metabolic activities [9]. This is because phosphorus plays a fundamental part in the production of energy and enzyme activation, in addition to being a structural element of nucleic acids and phospholipids and participating in processes such as cell division [8]. P deficiency generates significant impacts on commercially important crops, among them popcorn (*Zea mays everta*), causing large losses in yields.

The knowledge of the genetic basis regarding agronomic traits of interest is extremely relevant for plant breeding programs that seek to increase crop yield in areas where there is low soil-nutrient availability by means of generating superior hybrids and segregants for limiting conditions [10]. To this end, diallel crosses are widely used in cultivated species to provide genetic information by estimating the combining abilities of the parents and hybrids [11]. This strategy enables the estimation of the existence of additive effects from the parents and non-additive effects in crosses [12]. Diallel analysis, however, may become impractical depending on the number of lines to be used, requiring large experimental areas and labor in manual crosses. To solve this problem, the testcross method proposed by Davis [13] has been used as an option, allowing the evaluation of many lines in crosses with testers. By doing so, lines with inferior agronomic performance may be eliminated. As a result, the crosses show the most promising lines, thus making the procedure more effective [14].

Previous studies carried out for popcorn have proven the genetic action of the main traits of economic importance in the crop. In environments with adequate water and nutrient supply [15,16,17] and under water-deficit conditions [18,19] as well as P and N stress [10,20,21], additive genetic action prevails for grain popping expansion, while non-additive action has been the most important in the expression of grain yield under water stress [14,18,19]. These results are also consistent with studies conducted with biotic stressors [22,23,24,25,26].

Despite the relevance, only one research paper has been developed so far [10] toward understanding the influence of additive and non-additive effects on popcorn, a crop that earns about USD 1 billion annually in the United States. Given this, this study was considered relevant to contributing to filling the current gaps in knowledge regarding the genetic control of economically important traits for popcorn under phosphorus-limiting conditions. Therefore, the general and specific combining abilities and the genetic merit of 90 testcross hybrids of popcorn were estimated, and inferences were made about their additive and non-additive gene effects on the key agronomic traits of the crop. The goal was to propose breeding strategies for popcorn to develop cultivars adapted to phosphorus-limiting conditions as an option for leveraging Brazilian agribusiness.

## 2. Results

### 2.1. Genetic Variability for Agronomic Traits Evaluated under Contrasting Phosphorus Conditions in Both Environments

A significant difference between the genotypes (G) at a 1% probability level (*p* ≤ 0.01) was detected using the F test for all the traits (grain yield—GY, popping expansion—PE, and expanded popcorn volume—PV) for both levels of phosphorus availability, in Campos dos Goytacazes and Itaocara. The source of variation in phosphorus availability (P) and the G × P interaction also exhibited significant effects (*p* ≤ 0.01) for all the traits evaluated in the two locations studied (Table 1).

Regarding the effects of the general combining ability of the progenies (GCA I) and the testers (GCA II), the estimates were significant for all traits except for PV in Campos dos Goytacazes. Analyzing the mean square values for specific combining ability (SCA), it was observed that, for the GY, PE, and PV traits, there were highly significant values (*p* ≤ 0.01) in Campos dos Goytacazes. In Itaocara, there was only significance for GY at a 5% probability level (Table 1).

There was high significance for the GY and PE traits in Campos dos Goytacazes and for GY, PE, and PV in Itaocara regarding the progeny interaction with phosphorus availability (GCA I × P). The tester interactions with phosphorus levels (GCA II × A) and between specific combining ability and phosphorus levels (SCA × P) were found to be significant for all the traits evaluated.

### 2.2. General Combining Ability Effects

**Figure 1:**
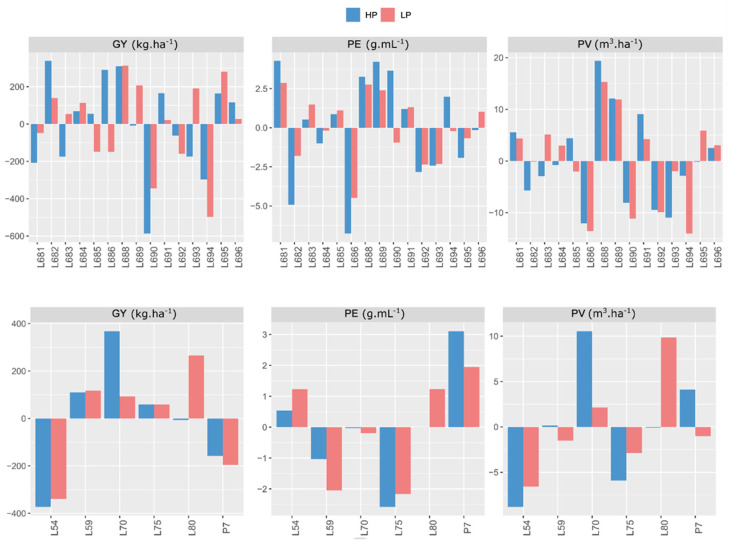
Estimates of general combining ability effects (*ĝi*) for three traits evaluated in 21 popcorn parents in a partial diallel scheme in Campos dos Goytacazes. HP—high phosphorus level; LP—low phosphorus level; PE—popping expansion; GY—grain yield; and PV—popcorn expanded volume per hectare; (−) signal indicating negative values.

General combining ability (GCA) estimates provide information about the additive effects of genes for the traits studied. From this, it may be stated that lines L682, L688, and L686, in Campos dos Goytacazes, showed the three highest values—338.431, 310.452, and 290.454, respectively—for GY in the environment without induced stress. In turn, for the low-phosphorus environment, the most positive values were assigned to the genotypes L688, L695, and L689 (313.311, 280.976, and 207.294, respectively) (Figure 1).

Considering the testers in question for GY, three—L70, L59, and L75—in the environment with high phosphorus levels and four—L80, L59, L70, and L75—in the environment with low phosphorus levels displayed positive deviations. Three of the best lines used as testers—L70, L59, and L75—presented positive values in both phosphorus conditions, and L59 was the tester that showed the lowest variation when comparing 367 to 739, ranking well in both high and low soil-phosphorus conditions in the Campos dos Goytacazes environment (Figure 1).

For the general combining ability effect for the PE trait, eight lines at the optimal phosphorus level and seven lines at the low phosphorus level showed positive values for PE, especially for L681, L689, and L690 at the optimal level and L681, L688, and L689 at the low P level (Figure 1). Lines L681 and L689 demonstrated the highest effects of GCA and the lowest variations when both environments were analyzed. Favorable performance was seen for the P7 and L54 lines in the environment with high phosphorus levels when evaluating the testers for PE. As for the phosphorus-deficient environment, the testers with positive values were P7, L80, and L54. Thus, the tester P7 achieved good performance regardless of the environmental conditions.

For the PV trait, the GCA estimates identified six lines—L688, L689, L691, L681, L685, and L696—with positive values in the environment with optimal phosphorus availability. On the other hand, in the environment with a low supply of the nutrient for this same trait, eight lines—L688, L689, L695, L683, L681, L691, L696, and L684—showed positive values. In the simultaneous analysis of the environments, the lines with the best results were L688 and L689, respectively. Additionally, considering the PV, of the six testers, three—L70, P7, and L59—expressed positive results in the environment with high phosphorus levels. In the environment with stress, only two testers—L80 and L70—had positive values. Among the genotypes with good results, tester line L70, from the BRS Angela population, showed positive values for PV at both phosphorus levels in Campos dos Goytacazes (Figure 1).

**Figure 2:**
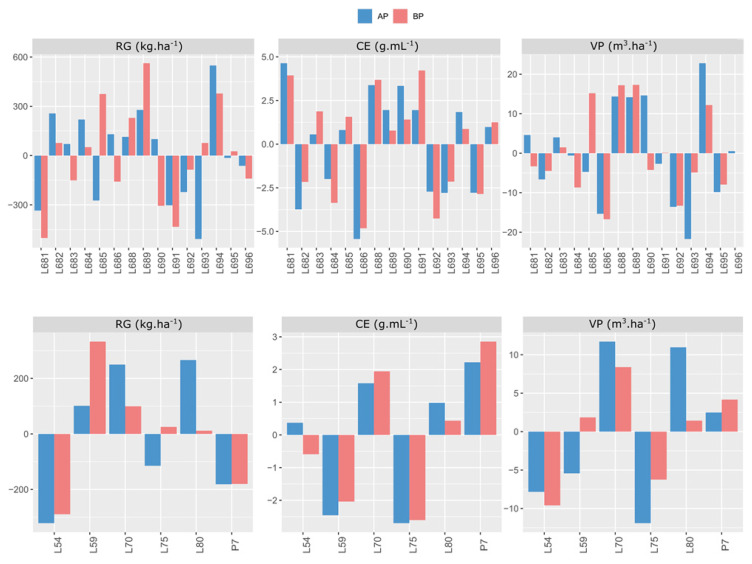
Estimates of general combining ability effects (*ĝi*) for three traits evaluated in 21 popcorn parents in a partial diallel scheme in Itaocara. HP—high phosphorus level; LP—low phosphorus level; PE—popping expansion; GY—grain yield; and PV—popcorn expanded volume per hectare; (−) signal indicating negative values.

The general combining ability effects for GY in Itaocara indicated that lines L694, L689, L682, L684, L686, L688, L690, and L683 had positive deviations from the mean in the environment with fertilizer recommendations. Under the nutrient-deficiency restriction, the best lines were L689, L694, L685, L688, L693, L682, L684, and L695. Given the best performances, lines L694, L689, L682, L684, and L688 were in both study environments. Thus, it should be stressed that lines L694 and L689 had the best positions in the ranking (Figure 2).

As for the *ĝi* effects of the testers for GY in Itaocara, three lines—L80, L70, and L59—showed positive values in the environment with satisfactory fertilization, while in the environment with induced stress, four genotypes—L59, L70, L75, and L80—were seen as the most relevant. It should be observed that, when analyzing the ranking of the two environments, tester L70 remained stable, ranking second, surpassed only by lines L80 and L59 in the environment with fertilizer recommendation and phosphorus restriction, respectively (Figure 2).

The evaluations conducted in Itaocara showed the following hierarchical ranking of the lines with positive values for the PE trait in the environment where phosphorus was applied: L681, L688, L690, L689, L691, L694, L696, L685, and L683. For low levels of phosphorus in the soil, however, there were changes in the performances of the lines, which began to be arranged according to the ranking of best performances, as follows: L691, L681, L688, L683, L685, L690, L696, L694, and L689 (Figure 2). Regardless of the interaction with the phosphorus levels, the genotypes expressing positive performance were the same. It is worth noting, however, that changes were caused in the ranking of the best genotypes, and that lines L681 and L688 displayed good stability, maintaining high positive values. Regarding the effect of the six testers studied, it may be stated that there was no change resulting from the environments, especially for P7, L70, and L80, in the expression of positive values (Figure 2).

The evaluations performed in Itaocara indicated that, for the trait of popcorn expanded volume per hectare (PV), lines L694, L690, L688, L688, L689, L681, L683, and L696; and L689, L688, L685, L694, L683, L691, and L696, were the most prominent in environments with optimal and low phosphorus levels, respectively. Among these, lines L694, L688, and L689 expressed positive values higher than 12.201 in a range from −21.73 to 22.80. This confirms the good performance of these lines when considering the effects of the general combining ability of the S_7_ progenies (Figure 2). The PV trait also allowed for discrimination of the testers L70, L80, and P7 in the environment with a phosphorus supply. As for the nutrient-deficient environment, the genotypes that expressed the highest positive values were: L70, P7, L59, and L80. Tester L70 showed good performance in both environments, with positive values of 11.713 and 8.391, respectively, when evaluated in Itaocara (Figure 2).

Considering the set of traits evaluated for *ĝi*, the lines with the best performance for GY and PE in the environment with high phosphorus availability in Campos dos Goytacazes were: L682, L688, and L686; and L681, L689, and L688, respectively. For the PV variable, the genotypes with the highest positive values were: L688, L689, L691, and L681. Of special relevance is the phenotypic plasticity of line L688, which exhibited positive values for all the variables, suggesting that there is a range of favorable alleles in this parent.

The best lines for GY in the stressed environment were L688, L695, and L689, and for PE, L681, L688, and L689. It is emphasized that lines L688, L689, and L695 ranked highest for PV. Particularly noteworthy is the good positive deviation performance of L688 and L689 in the nutrient-deficient environment, confirming the presence of favorable alleles for phosphorus use efficiency in the parents from the UENF-14 population (Figure 1 and Figure 2).

When the set of testers was evaluated, it can be seen that, for GY and PE, the genotypes L70 and L59, and P7 and L54, respectively, had good performances in the environment with the optimal phosphorus level in Campos dos Goytacazes. Regarding the PV trait, the most prominent genotypes were L70, P7, and L59, as they showed the highest positive values. In the environment with low phosphorus levels, a change in the performance of the testers was observed, in which the highest positive values were seen for lines L80, L59, and L70 relative to the GY trait. The most relevant genotypes for PE were P7, L80, and L54. The PV allowed tester lines L80 and L70 to stand out in presenting the highest positive values.

When comparing the individual values of GY and PE for the PV trait in relation to the experiments carried out in Itaocara, the genotypes L694, L690, and L688 gave the best performances. The order of the most positive values for variables GY and PE was attributed to lines: L694, L689, L682; and L681, L688, L690, respectively. Based on the considerations, line L688 showed good performance for both PV and PE and is considered a promising genotype. Regarding the environment with low phosphorus levels, the lines with the best performances were L689, L694, and L685 for GY; and L691, L681, and L688 for PE. Considering the PV, the genotypes with the highest positive values were L689, L688, and L685 (Figure 1 and Figure 2).

Upon analyzing the testers when grown in Itaocara, differences in performance could also be identified. The most prominent positive values were found in L80, L70, and L59 for GY; and in P7, L70, and L80 for PE in the environment with the proper adjustments for phosphorus. Considerations carried out for PV allowed us to indicate the parents L70, L80, and P7 as the most promising. For the nutrient-deficient condition, the variable PV enabled us to distinguish lines L70, P7, and L59 as having the most positive values. For GY and PE, the genotypes L59, L70, and L75; and P7, L70, and L80, respectively, were the most relevant in this same environment.

### 2.3. Genetic Merit of Hybrids

**Figure 3:**
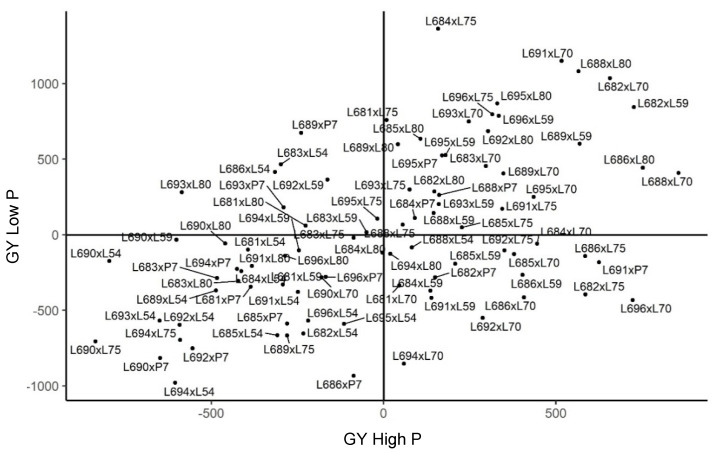
Estimates of the genotypic value (${\hat{s}}_{ij}$) of hybrids for grain yield (GY) in diallel hybrids of popcorn evaluated under high and low phosphorus availability in Campos dos Goytacazes. (−) signal indicating negative values.

Specific combining ability effects (SCA—${\hat{s}}_{ij}$) are the result of the presence of dominance gene effects. The effects of ${\hat{s}}_{ij}$ for GY in Campos dos Goytacazes showed 46 hybrids with positive variances, suggesting that the dominance gene effects prevail, which expressed amplitudes from 2.289 to 616.921 in the environment with phosphorus supplementation. The three hybrid combinations that exhibited the highest heterosis, considering ${\hat{s}}_{ij}$ for this trait, were L691 × P7, L686 × L80, and L689 × L59. Among the testers, the parents L75, L54, and L80 were involved in most crosses with positive SCA values, with magnitudes of 21.73%, 17.39%, and 17.39%, respectively (Figure 3).

On the other hand, in the environment with induced stress, the number of genotypes with positive values for ${\hat{s}}_{ij}$ was significantly reduced. Among the 90 hybrids tested, only 37 crosses expressed positive magnitudes, which ranged from 2.601 to 1189.951. The most vigorous genotypes for ${\hat{s}}_{ij}$ were L684 × L75, L691 × L70, and L686 × L54. As for the highest percentages of crosses with positive estimates in this case, they occurred with the testers L80 and L59, with values of 21.62% and 18.91%, respectively (Figure 3).

Therefore, the selection of parents based on additive genetic merit in Campos dos Goytacazes enabled the identification of genotypes with genetic potential to be phenotypically superior for each environment. In the experiment with fertilizer adjustments, the highest genetic merits were expressed in the combinations L88 × L70, L682 × L59, and L696 × L70, whereas in the nutrient-deficient environment, the hybrids L684 × L75, L691 × L70, and L688 × L70 expressed the highest genetic merits, exhibiting good allelic complementation (Figure 3).

**Figure 4:**
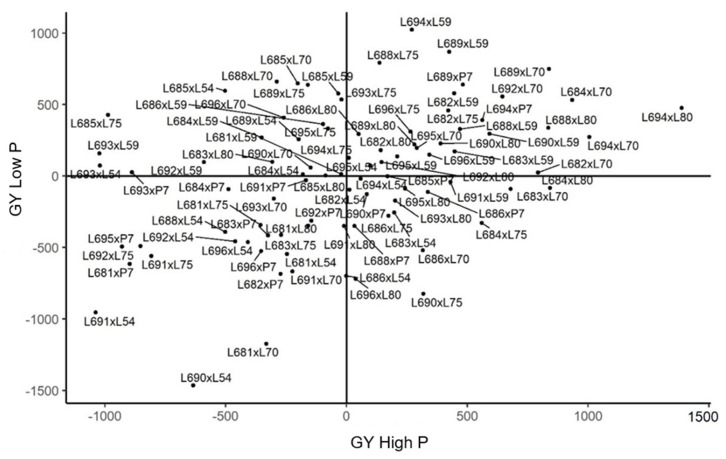
Estimates of the genotypic value (${\hat{s}}_{ij}$) for grain yield (GY) in diallel hybrids of popcorn evaluated under high and low phosphorus availability in Itaocara. (−) signal indicating negative values.

The experiments conducted in Itaocara showed that the effects of ${\hat{s}}_{ij}$ enabled 49 hybrids to be discriminated, with heterosis values estimated from 22.423 to 631.804 in the environment with fertilization recommended for the popcorn crop. This selection indicated the crosses with the highest dominance gene effects, with the hybrids L691 × L59, L685 × P7, and L692 × L70 standing out as having the best performances. Regarding the estimates of the general combining ability, the testers L80, L70 and L59 showed positive values, being in the formation of the best genotypes. Among them, line L59 was the most frequent, present in 20% of the best hybrid combinations (Figure 4).

In the experiments for GY carried out in Itaocara, there was no considerable reduction in the number of genotypes obtained in the environment with nutrient reduction. Thus, from 90 combinations, 51.10% of the crosses had positive values. Among them, three hybrids (L691 × P7, L692 × L70, and L688 × L75) stood out with high estimates of ${\hat{s}}_{ij}$, and the greatest dominance gene effect was shown by the combination L691 × P7, representing high allelic complementation in the parents. The most frequent tester in the crosses was line L80, as it participated in nine positive crosses, accounting for approximately 20% of the positive combinations in Itaocara. Analyzing the *ĝi* estimates of this tester, the additive effect of the genes is evident, since it showed positive estimates and high magnitudes in the nutrient-deficient environment (Figure 4).

From the estimates of additive genetic merit, it was observed, in the experiments conducted in Itaocara, that the values of GY emphasized the hybrids L694 × L59, L689 × L59, and L88 × L75 as having the highest estimates of heterosis when grown in an environment with nutrient stress. In the environments with added phosphorus, the best allelic complementation was found in L694 × L80 and L694 × L70 (Figure 4).

**Figure 5:**
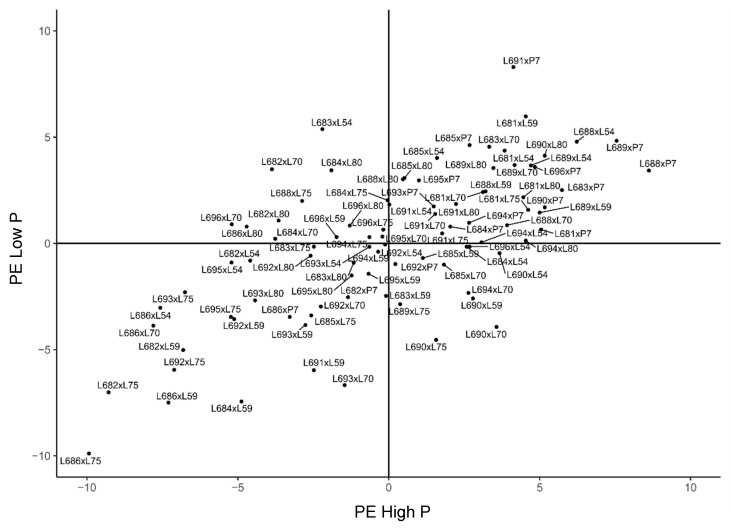
Estimates of the genotypic value (${\hat{s}}_{ij}$) for popping expansion (PE) in diallel hybrids of popcorn evaluated under high and low phosphorus availability in Campos dos Goytacazes. (−) signal indicating negative values.

In relation to the PE variable, the evaluations conducted in Campos dos Goytacazes under conditions with phosphorus supplementation made it possible to point out the testers L59 and L70 due to their positive SCA values and the highest frequencies in the formation of hybrids with positive estimates of ${\hat{s}}_{ij}$, corresponding to 20% of the crosses. These parents, however, showed no additive gene effects for their *ĝ_i_* estimates. Additionally, for PE, 50% of the hybrid combinations showed positive magnitudes of ${\hat{s}}_{ij}$. However, the values showed a low range, varying from 0.166 to 3.534. The hybrids that exhibited the highest heterosis for SCA were L684 × L75, L691 × L75, and L684 × L54, respectively (Figure 5).

In the low-phosphorus environment, it was possible not only to obtain 47 hybrids with high gene complementation, in which the SCA estimates ranged from 0.053 to 5.483, but also to detect that the crosses L682 × L70, L681 × L59, and L691 × P7 had the highest SCA values. In terms of tester performance, line L59 had the greatest participation in the formation of superior hybrids, being the source of 21.27% of the best combinations (Figure 5).

When the genetic merit of the hybrids for PE was evaluated in both growing conditions in Campos dos Goytacazes, it was found that the hybrid combinations with the highest genetic merit were L691 × P7, L683 × L54, and L681 × L59 in a low-phosphorus environment, and L688 × P7, L689 × P7, and L688 × L54 in an environment with phosphorus supplementation (Figure 5).

**Figure 6:**
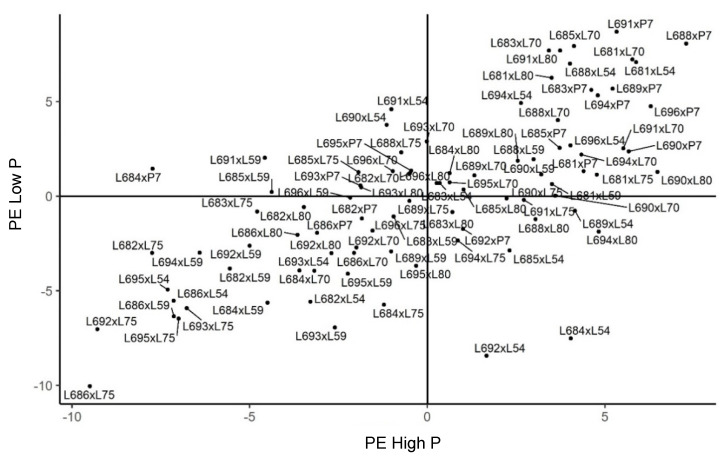
Estimates of the genotypic value (${\hat{s}}_{ij}$) for popping expansion (PE) in diallel hybrids of popcorn evaluated under high and low phosphorus availability in Itaocara. (−) signal indicating negative values.

In Itaocara, the experiments for PE inferred that the tester P7 had the highest percentages of allelic complementation in the crosses, with high estimates of ${\hat{s}}_{ij}$ for 22% of the hybrid combinations in the phosphorus supply condition, and 20.90% in the nutrient-deficient environment. Thus, based on the combining abilities, it can be stated that the tester P7 has genes favorable for phosphorus use efficiency, and also expresses more pronounced genetic divergence than the other parents (Figure 6).

Fifty percent of the 90 hybrids evaluated for ${\hat{s}}_{ij}\;$ displayed crosses with dominance gene effects in the environment with optimal phosphorus availability, whose combinations with the highest expressions of heterosis were: L684 × L54, L692 × L54, and L684 × L7. As for the environment with low phosphorus level, 47.80% of the combinations exhibited positive values. Of these, the hybrids that performed best were L694 × L54, L685 × L70, and L684 × L80, with the highest values of the specific combining ability effects (Figure 6).

Regarding the genetic merit of the hybrids tested in the environments, it was found that the combination L688 × P7 presented favorable alleles for the highest expression of PE, in addition to having good phenotypic plasticity, since it ranked well in both growing conditions. Among the hybrids with the best performance in the environment with phosphorus supplementation, the following combinations can be considered: L688 × P7, L696 × P7, and L690 × L80. As for the environment without a phosphate fertilizer supply, the hybrids ranking highest were L91 × P7, L685 × L70, and L688 × P7 (Figure 6). The good performance of the tester P7, which was in the best hybrid combinations and expressed the highest *ĝi* estimates in both high and low P level environments, should again be noted.

**Figure 7:**
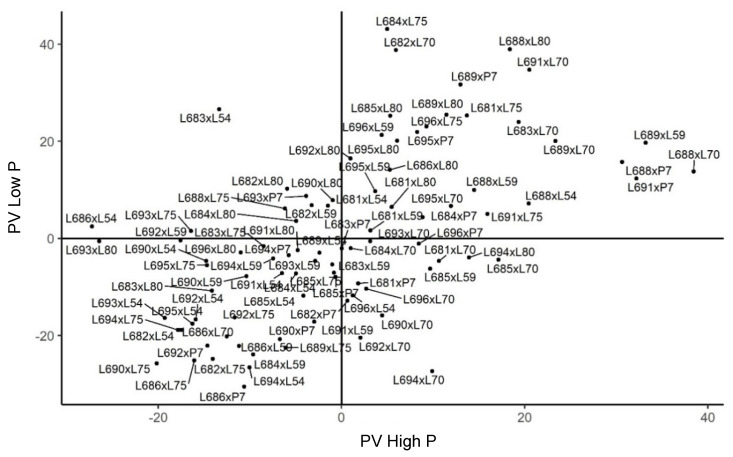
Estimates of the genotypic value (${\hat{s}}_{ij}$) for expanded popcorn volume per hectare (PV) in diallel hybrids of popcorn evaluated under high and low phosphorus availability in Campos dos Goytacazes. (−) signal indicating negative values.

In the evaluation of PV in Campos dos Goytacazes, 56.70% of the combinations were found to have positive estimates of ${\hat{s}}_{ij}$ in the environment with phosphorus supplementation. The combinations L689 × L59, L691 × P7, and L686 × L80 expressed the highest estimates of dominance gene effects. Such results corroborate the effects seen for GY when its SCA was evaluated. This result is understandable, since PV is a combination of GY and PE (Figure 7).

Regarding the best tester for this index, line L70 provided the highest heterosis in the crosses, totaling 21.56% of the combinations with positive values. This tester, along with line L80, showed the highest additive gene effects of *ĝ_i_* for this trait. In the environment without the nutrient supplementation of phosphorus, the most frequent tester in the crosses with good allelic complementation was the parent L80 when evaluating its SCA estimates, which comprised 25% of the crosses with higher magnitudes of SCA. This result corresponds with the additive gene effects assigned to this genotype when its *ĝ_i_* estimate was analyzed. As for the hybrids with the highest SCA estimates, they were L684 × L75, L682 × L70, and L691 × L70, respectively, when considering the 36 crosses with the highest magnitudes of dominance effect (Figure 7).

The genetic merit of the hybrids was also quantified for the PV index of plants grown in both environments in Campos dos Goytacazes. The evaluations indicated that the most vigorous combinations corresponded to L684 × L75, L682 × L70, and L688 × L80, in the nutrient-stressed environment, and L688 × L70, L689 × L59, and L691 × P7, in the experiment with a phosphorus supply. It should be noted that line L688 and tester L70 exhibited excellent performances per se when analyzing their respective general combining abilities, corroborating the prevalence of additive gene effects in both gene conformations (Figure 7).

**Figure 8:**
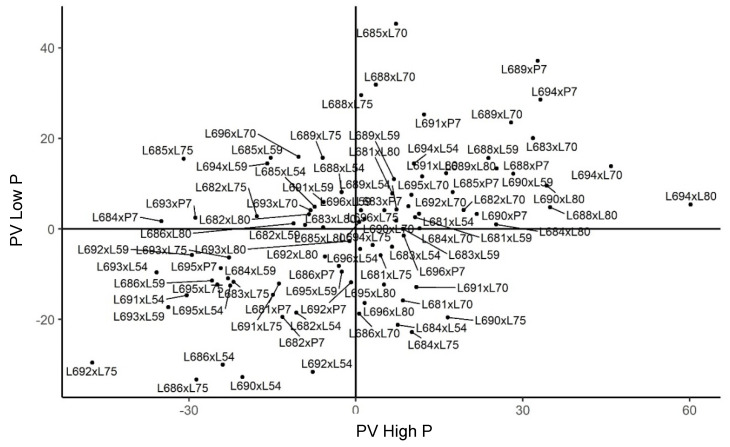
Estimates of the genotypic value (${\hat{s}}_{ij}$) for expanded popcorn volume per hectare (PV) in diallel hybrids of popcorn evaluated under high and low phosphorus availability in Itaocara. (−) signal indicating negative values.

The PV supercharacter also allowed for the discrimination of the behavior of the 90 hybrids evaluated in Itaocara. The combinations L694 × L80, L684 × L75, and L685 × P7 stood out with the highest heterosis in a set of 49 hybrids, for which there was a prevalence of dominance gene effects in the environment with phosphorus supplementation. The most heterotic hybrid—L694 × L80—is assumed to have the highest divergence among the parents (Figure 8).

In the low-phosphorus environment, 47 hybrids expressed dominance gene effects, with their amplitudes ranging from 0.255 to 21.709 for estimates of ${\hat{s}}_{ij}$. After selecting the hybrids with positive values, the ones with the highest heterosis were determined: L685 × L70, L691 × P7, and L688 × L75, respectively. It should be mentioned that tester L70, which comprised the best hybrid, was also found in a greater number of positive combinations for both environments (Figure 8). Therefore, it may be assumed that the higher frequency of this parent may be related to the additive gene effects attributed to it when considering its *ĝ_i_* estimate.

The evaluation of the combining abilities for PV allowed us to know the genetic merit of the hybrids when grown in Itaocara. This analysis pointed out that the combinations L694 × L80, L694 × L70, and L688 × L80 had the best performances for the high-phosphorus-availability conditions. Hybrids L685 × L70, L689 × P7, and L688 × L70 were also identified as the ones with the best performance in the low-phosphorus environment. Moreover, using the evaluations conducted for PV, it could be verified that there was a predominance of the L70 and L80 testers in the low- and high-nutrient-availability environments. Additionally, the combinations L689 × P7 and L694 × P7 presented the greatest phenotypic plasticity since their recommendations may be made in either an environment with low or high phosphorus levels (Figure 8).

## 3. Discussion

The genetic variability among the lines under study due to the significant differences among the genotypes for the traits investigated—GY, PE, and PV—in Campos dos Goytacazes and Itaocara is evident (Table 1). Furthermore, the source of variation in phosphorus (P) availability also had significant effects for all the traits; this suggests that the dose of phosphorus applied to the soil was adequate to provide a distinction between the environments and enable correct differentiation of the efficient genotypes from the inefficient ones in phosphorus use in Campos dos Goytacazes and in Itaocara. This points out that the line classification differed between the two growing conditions (high and low phosphorus levels in the soil), indicating a specific P-related effect. This provides strong evidence that there was significant variation in the ability of the material investigated to explore P in its environment, a prerequisite for selection-based breeding. However, screening this performance is not informative with respect to the number of genes underlying the variation observed, but encourages further research focusing on QTL analysis to provide more information on the genetic architecture of variation in response to different P levels in soil [27].

In addition to this, genetic variability in germplasm collections is an essential factor in obtaining genetic gains in breeding programs. Due to the high consumption of phosphate fertilizers—which may lead to a shortage of P reserves within a few decades—and the need to develop sustainable agriculture, these genotypes have become important sources of tolerance for breeding programs of popcorn to obtain gains in efficiency and responsiveness in phosphorus use. Gerhardt et al. [10] also reported genetic variability for agronomic traits when evaluating popcorn hybrids and lines at contrasting phosphorus levels in soil.

The G × P interaction, in turn, was significant for all the traits, suggesting a dissimilar response of the popcorn hybrids under different phosphorus-availability conditions (Table 1). This interaction may provide changes in the classification of hybrids between the experiments with high and low phosphorus levels. In this regard, Vencovsky and Barriga [28] recommend that the practice of genotype selection should be environment-specific, that is, each environment should have a set of distinct or partially distinct genotypes. Thus, selection should not be made based on average performance, as favorable alleles that control the expression of the character under stress differ, at least partially, from favorable alleles that control the same character under optimal conditions [29].

Significance in the estimates for all variables, except for PV, was observed in Campos dos Goytacazes when the effects of the source genotype variation were unfolded into general combining ability (GCA) and specific combining ability (SCA). This demonstrates that by exhibiting significance for GCA and SCA, GY and PE showed variability resulting from additive and non-additive effects in controlling gene expression. The significance of variation attributed to the additive effects proves that there are promising parents to be used, mainly in intrapopulation breeding programs, as this variation is very useful in pre-breeding to incorporate exotic germplasms into adapted populations or to adapt populations to abiotic stressors (Table 1). The magnitude of the additive variance expressed by the mean squares of the GCA of the progenies and the testers indicates the existence of significant additive effects of the genes [28].

Regarding the effects of specific combining ability—highly significant for the variables GY, PE, and PV (*p* ≤ 0.01) in Campos dos Goytacazes—it may be assumed to be the gene action of dominance in the expression of PE, which is a trait known to be governed by additive effects, as reported by Dofing et al. [30], Pacheco et al. [31], Larish and Brewbaker [32], and Pereira and Amaral Júnior [15]. As for Itaocara, there was only significance for GY at a 5% probability level (Table 1). This indicates that non-additive gene effects also exert an influence on these traits and suggests possible allelic complementation between the parents at the respective loci, with some degree of dominance. This possibility for PE gene expression agrees with more recent results obtained by Coan et al. [33], who support the existence of mixed inheritance in the expression of this trait.

In view of the significant progeny interaction with contrasting phosphorus levels (GCA I × P) for the traits GY and PE in Campos dos Goytacazes, and for the traits GY, PE and PV in Itaocara, it may be assumed that the additive gene effects between the lines provided differences. In this case, then, selection for each level of phosphorus is recommended, as already mentioned. The tester interaction with phosphorus levels (GCA II × P), also significant for all the traits evaluated, suggests that selection should be made for each phosphorus level. Gerhardt et al. [10], likewise, found significance for the interaction between popcorn progenies and contrasting environments in using phosphorus.

From the results, considering the significant interaction between specific combining ability and phosphorus levels (SCA × P), it can be noticed that the classification of SCA effects differed between environments. The results at each fertilization level should, thus, be considered to allow for the effective selection of hybrids that are efficient and responsive to phosphorus use.

As for the experimental precision expressed by the coefficient of experimental variation (CVe), the values found for all the traits were less than 20%, suggesting excellent experimental precision [34]. These results agree with the estimates found by Santos et al. [35] (2017) and Gerhardt et al. [10] in experiments with abiotic stressors in popcorn.

According to Sprague and Tatum [36], informed by Inocente et al. [37], the GCA corresponds to the average behavior of a line in a series of hybrid combinations, and is expressed by the *ĝi* estimate. For Cruz and Vencovsky [38], a low value of *ĝi* suggests that the average of the hybrids in which line *i* participates does not differ much from the overall average of the diallel, meaning that when there are high values, positive or negative, parent *i* is superior or inferior to the other parents in the diallel when compared to the average of their hybrids. Thus, the estimates of the *ĝi* effects for the variables analyzed have values with signs ranging from negative to positive as a function of the performance of the parent. Accordingly, Cruz and Vencovsky [38] and Scapim et al. [39] affirm that the line with the highest frequency of favorable alleles will express a higher *ĝi*.

Based on these references and considering the *ĝi* estimates, it is observed that, in Campos dos Goytacazes, lines L682, L688, and L686 had the three highest values—338.431, 310.452, and 290.454, respectively—in the environment with adequate phosphorus availability, and, in the environment with low phosphorus levels, the most positive values were assigned to the genotypes L688 (313.311), L695 (280.976), and L689 (207.294) (Figure 1).

Line L688 was not affected by a substantial reduction in phosphorus in the soil; thus, its high combining ability and phenotypic stability are relevant for programs aimed at increasing GY. Scapim et al. [39] reported the use of popcorn populations with high GCA estimates to form 211 varieties with high GY and PE, supporting the relevance of working with parents with high *ĝi* estimates for these traits, which are both of major relevance for popcorn trading.

Regarding the testers, for GY, under optimal phosphorus supply conditions, three lines showed positive deviations (L70, L59, and L75) and four stood out for the environment with induced stress (L80, L59, L70 and L75). Among the best lines used as testers, three—L70, L59, and L75—had positive values in both conditions of phosphorus availability. It should be emphasized that tester L59 had the least variation (367 and 739), ranking well in both phosphorus conditions in the soil, in Campos dos Goytacazes. This good performance may be associated with its genealogy (Beija-flor: UFV) and adaptation to a temperate/tropical climate, a factor correlated with the presence of favorable alleles in a good parent (Figure 1).

For PE, eight lines under optimal conditions of phosphorus availability and seven lines under limiting conditions of the nutrient showed positive values for *ĝi* estimates in Campos dos Goytacazes. There is agreement in the prominence of lines L681, L689, L690, L681, L688, and L689 (Figure 1) in both conditions of phosphorus availability; however, lines L681, and L689 had the greatest *ĝi* effects and least variation between the two environments. Therefore, it may be recommended that these parents obtain lines with good PE, since *ĝi* is the estimator that indicates the parents with the best average performance in crosses. These lines, thus, have the highest concentrations of favorable alleles for traits predominantly influenced by additive gene action.

As for the testers that were outstanding in environments with high (P7 and L54) and low (P7, L80, and L54) phosphorus levels, P7 showed the best performance regardless of phosphorus availability in the soil; this represents a line of interest to be included in crosses aimed at obtaining superior hybrids in relation to efficiency and responsiveness in phosphorus use.

Kamphorst et al. [40] further reported that line P7 showed high GY grain yield averages when grown in a water-stressed environment, and were considered agronomically efficient in water use. This suggests that this line has mechanisms to withstand adverse situations imposed by abiotic stress. Santos et al. [20] also described the relevance of P7 for GY and PE traits under optimal and low soil-nitrogen-availability conditions from a panel of ten popcorn lines evaluated in Campos dos Goytacazes and Itaocara. When evaluating 25 popcorn lines under high and low P conditions in soil, Gerhardt et al. [10] pointed out that, because of high *ĝi* estimates for GY and PE, line P7 has high potential for obtaining superior hybrids in terms of efficiency and responsiveness in phosphorus use.

In Campos dos Goytacazes, six lines distinguished themselves, for PV, by having positive values of *ĝi* estimates in the environment with high phosphorus levels—L688, L689, L691, L681, L685, and L696—and, in limiting conditions of the nutrient, eight lines stood out with positive values, namely: L688, L689, L695, L683, L681, L691, L696, and L684. When both environments were analyzed, it was noted that the lines with the best performance were L688 and L689, respectively (Figure 1). This coincidence of results corroborates the good performance of these genotypes and the substantial incidence of favorable alleles for phosphorus efficiency and use in the lines derived from the UENF-14 population (Table 1). The PV trait is considered a supercharacter that is intended to simulate a selection index, which enables concomitant gains for the two main traits of economic importance—GY and PE expansion—for the popcorn crop [41].

Still considering the PV, among the testers investigated, lines L70, P7, and L59 prevailed in the environment of high phosphorus levels in the soil, having shown positive *ĝi* results, whereas in phosphorus-limiting conditions, only two stood out—L80 and L70. Among the genotypes with good results, tester line L70, from the BRS Angela population, distinguished itself by having positive values for PV at both phosphorus levels in Campos dos Goytacazes (Figure 1). These results agree with the work of Schmitt et al. [42], who reported the good performance of the parent L70 in presenting a positive *ĝi* estimate for PE.

As for the effects of GCA for GY, in Itaocara, eight lines were highlighted based on *ĝi* estimates in the environment with high phosphorus level. Similarly, in low P conditions, eight lines also stood out. Considering both conditions, however, five lines distinguished themselves in both environments by presenting positive deviations, as follows: L694, L689, L682, L684, and L688. Within them, lines L694 and L689 were highlighted in the ranking, in that order (Figure 2).

When it comes to the *ĝi* effects of the testers, still for GY in Itaocara under high phosphorus conditions, three lines were prominent, and four genotypes were superior under P-deficient conditions in the soil. When analyzing both conditions of phosphorus availability, tester L70—which has adaptations to tropical environments—displayed greater stability, only surpassed by lines L80 and L59—adapted to temperate and tropical climates—which were superior, respectively, in high- and low-P environments. This suggests good adaptation of these genotypes to the conditions studied for GY. By evaluating 15 populations of popcorn from different Latin-American countries under water-stress conditions, Santos et al. [43] related the importance of varieties with temperate and tropical climatic adaptations. Thus, according to the authors, the relevance of these materials in studies with popcorn to obtain superior genotypes is reinforced. Gerhardt et al. [10] pointed out, in a previous study, line L59 as being efficient and responsive in the use of phosphorus. Therefore, it is a genotype that expresses superior yields to the averages in environments of high and low phosphorus levels.

In this context, it is verified that, for the best S_7_ progenies, as well as for the testers, the *ĝi* estimates for PE were negative. This is due to the negative genetic correlation between GY and PE, a phenomenon already reported in other research conducted in the popcorn crop [15,16,30,43,44,45,46,47]. This correlation, therefore, makes it difficult to obtain genotypes with high GY and high popping expansion concomitantly [15,48,49].

By analyzing the results of the *ĝi* estimates for PE in Itaocara, even with the complex interaction, the best-performing genotypes, under optimal phosphorus conditions in the soil, were also better under limiting conditions. In this case, there were only differences in the ranking order, indicating that the change in the crop environment caused alterations in the performance of the genotypes under different P conditions. As stated before, when the interaction is of the complex type, it makes it difficult to indicate genotypes for a group of environments, requiring the breeder to recommend appropriate genotypes for specific conditions. The performance of lines L681 and L688 proved to be encouraging for this study, since they showed good phenotypic plasticity for the trait in question, ranking well in both environments, regardless of the P supply. Regarding the testers, the positive results of lines P7, L70, and L80 in both environments distinguish them as potential candidates for obtaining superior hybrids in both environments.

With regard to the *ĝi* effects for PV in Itaocara, lines L694, L688, and L689 were superior to the others as they showed positive values under optimal phosphorus conditions and when under nutrient stress, not exhibiting significant differences in performance, and keeping the positive values for *ĝi*. For the testers, L70 showed the same behavior, performing well under both phosphorus-availability conditions.

Given these considerations, it may be stated that the UENF-14 popcorn population has a high frequency of alleles favorable for the efficiency of phosphorus use in the soil. This assertion is supported by the good performance of lines L688 and L689, which showed good general combining ability and high GY, as well as high popping expansion, suggesting that there is a distinct heterotic pattern. Thus, lines L688 and L689 and testers P7 and L59 are highly recommended for use in future hybrid combinations aimed at increasing GY and PE in environments with low soil-phosphorus levels in breeding programs focusing on more sustainable agriculture.

By considering the merit of the hybrids for the GY and PV traits together, it was assumed that the L688 × L70 cross had higher heterosis when evaluated in environments with high P levels in the soil. In the environment with stress induction, heterosis was more evident in the genotype L684 × L75. Therefore, the interaction of these genotypes with the environments provided relative superiority compared to the others, suggesting that there are favorable alleles in these hybrid combinations.

For the traits PE and PV together, changes in the ranking of the crosses were observed, highlighting the superiority of the genotype L689 × L59, which showed the highest heterosis in the environment with high phosphorus levels. Thus, the hybrid combination L689 × P7 may be considered the most relevant one for the low-phosphorus environment in the experiments in Campos dos Goytacazes. As for Itaocara, the hybrid combinations with the highest popping expansion were L694 × L80 in the environment with added phosphorus, and L685 × L70 in the environment with low phosphorus levels. In relation to the testers, lines P7 and L54 showed additive genetic effects for GCA in the deficient environments, increasing popping expansion. The other testers distinguished themselves through their good allelic complementation in the crosses in which they participated.

When considering GY and PV traits together in Itaocara, the hybrid L694 × L80 was chosen as the most prominent for the environment with fertilizer recommendation for popcorn. For the low-phosphorus environment, the combination with the highest heterosis was L685 × L70; this hybrid also showed positive a magnitude of ${\hat{s}}_{ij}$ when its SCA was evaluated.

More generally, the genotypes utilized present genetic potential for obtaining hybrids with high heterosis for efficiency and responsiveness in phosphorus use. Considering the prevailing gene effects of the GY, PE, and PV traits in the contrasting environments, the hybrid combinations that displayed promising results for efficiency and responsiveness in phosphorus use were L688 × L70, L694 × L80, L688 × L70; L694 × L80, L689 × L59, L694 × L80; and L689 × P7, and L689 × L59, respectively.

From the information obtained by means of the combining ability and having the most promising combinations, the parents from the UENF-14 population with the best performance—L688, L694, and L689—may be selected as standards for the creation of a new heterotic group and as testers of new lines from this heterotic group. Thus, this new heterotic group should be maintained and used separately for the generation of variability within the group, and for the selection of new lines.

Accordingly, the implementation of a procedure called “line recycle”, originating from the UENF-14 population, is also proposed; this will form new biparental populations directly or via backcrossing, in which the elite lines L688, L694, and L689 from the UENF popcorn breeding program will be crossed among themselves, in subsequent cycles, within this same heterotic group. Thus, new lines will be obtained with superior traits to their parents because of the increased frequency of favorable alleles and the consequent higher level of heterosis when crossed to form a heterotic group.

In conclusion, to meet the agroeconomic aspects of the North and Northwest regions of Rio de Janeiro State—which tend to expand—on the basis of the combining ability and of the presence of favorable alleles, we propose obtaining a triple hybrid derived from a simple hybrid from parents originating from the heterotic group of the UENF-14 population—L688 × L689, for example, whose parents showed high GCA; this will be used as the female parent in a cross with the L80 line. Such a line has been demonstrated to be sufficiently vigorous to ensure good pollination and, consequently, satisfactory GY in female plants.

## 4. Materials and Methods

### 4.1. Plant Material, Experimental Design, and Environmental Conditions

Six S_7_ lines from the Germplasm Bank of the *Universidade Estadual do Norte Fluminense Darcy Ribeiro* (UENF)—previously classified by Gerhardt et al. [50] regarding P use—were used as testers, of which three (L59, L70, and P7) were efficient and responsive and three (L54, L75, and L80) were inefficient and non-responsive. They were used as testers in crosses with 15 other progenies (L681, L682, L683, L684, L685, L686, L688, L689, L690, L691, L692, L693, L694, L695, and L696) (Table 2) from the UENF-14 open-pollinated variety. This population was selected after five cycles of intrapopulation recurrent selection, adapted to the soil and climate conditions of the North and Northwest regions of Rio de Janeiro State [51]. Ninety testcrosses were generated from the crosses in a partial diallel scheme of the six testers with the 15 UENF-14 progenies.

The experiments were conducted in a randomized block design with replication arrangements within sets. Five sets of 18 treatments (15 progenies plus three controls: UENF N 01, UENF N 02, and UENF HS 03) in each set were utilized, with three replications. These experiments were carried out during the harvest period, between October 2019 and March 2020, in two locations and under two contrasting conditions with respect to phosphorus availability (high and low phosphorus). The sites were the Experimental Station of the Colégio Estadual Agrícola Antônio Sarlo, in the municipality of Campos dos Goytacazes (latitude: 21°42′48″ S, longitude: 41°20′38″ W, 14 m above sea level), and the Experimental Station of Ilha do Pomba, in the municipality of Itaocara (latitude: 21°38′50″ S, longitude: 42°03′46″ W, 58 m above sea level); these correspond, respectively, to the Northern and Northwestern regions of Rio de Janeiro State. The climate in Campos dos Goytacazes and Itaocara is classified as humid tropical (Aw), with hot summers and mild winters, with rainfall tending to be concentrated in the summer months.

Sowing was performed according to the conventional planting system, with a stand of 15 plants per plot, or 55,555 plants per hectare. Each experimental plot consisted of a 3.00 m row with 0.90 m spacing between rows and 0.20 m spacing between plants.

Prior to the experiments, soil chemical analysis was conducted to characterize the environments in terms of nutrient availability from samples collected in the 0–10 and 10–20 cm layers, forming a sample composed of ten subsamples (Table 3). The available P content was determined using the Mehlich-1 extractor. According to the clay soil content of Campos dos Goytacazes and Itaocara, the phosphorus levels were classified as low [52] (Ribeiro et al., 1999).

Two doses of this fertilizer were used to simulate contrasting environments and to stimulate the genotypes to express the genes responsible for phosphorus efficiency and responsiveness. In the environment with high phosphorus availability, 30 kg ha^−1^ of N (in the form of urea), 85 kg ha^−1^ of P_2_O_5_ (triple superphosphate), and 40 kg ha^−1^ of K_2_O (potassium chloride) were applied. For the environment with low phosphorus availability, 30 kg ha^−1^ of N, 0 kg ha^−1^ of P_2_O_5_, and 40 kg ha^−1^ of K_2_O were used, which means the fertilizer in the stressed environment did not contain phosphorus. Topdressing fertilization was performed in partial applications in both environments when the plants reached the phenological stage of four (V4) and six (V6) fully expanded leaves at a concentration of 100 kg ha^−1^ of N (in the form of urea). The other phytosanitary treatments were performed according to the recommendation for the crop in the North and Northwest regions of Rio de Janeiro State [53]. The experiments received additional irrigation whenever necessary to avoid water stress.

### 4.2. Evaluated Traits

The following traits were evaluated: (i) Grain yield (GY)—expressed by the average grain yield of the experimental unit in grams per plot, adjusted to 13% moisture and extrapolated to kg ha^−1^. (ii) Popping expansion (PE)—obtained using the ratio between the expanded popcorn volume and the mass of 30 g, expressed in mL g^−1^, utilizing the average of two samples per plot. Popping was conducted in a microwave oven, with 1200 Watts of power for 2 min, and the expanded popcorn volume was quantified in a 2000 mL beaker. The resulting value was divided by the initial weight of the 30 g grain, and the final result expressed in mL g^−1^. (iii) Expanded popcorn volume per hectare (PV), determined using the product between GY and PE, with the final value divided by 1000 and expressed in m^3^ ha^−1^.

### 4.3. Data Analysis

Adjustments of the set effects for all the hybrids were made according to the average of the controls common to all the sets before conducting the analysis of variance. We estimated the average of the controls for each set (ACS) and the general average of the controls (GAC); using the GAC/ACS relationship, the adjustment factor for each set was obtained, following the procedure used by Ribeiro et al. [54] and Guimarães et al. [55].

After these adjustments, the analyses were performed following the randomized block model. At first, the individual analyses of variance were conducted considering the environments with high and low phosphorus, according to the following statistical model: Y_ij_ = m + B_j_ + G_i_ + e_ij_, in which Y_ij_ is the observation of the i-th genotype in the j-th block; m is the general constant; B_j_ is the effect of the j-th block; G_i_ is the effect of the i-th genotype; and e_ij_ is the experimental error associated with the observation Y_ij_, which is normally and independently distributed (NID—0, *σ*^2^).

Subsequently, a joint analysis of variance was conducted to determine possible interactions between the genotypes and the levels of phosphorus in each location. The joint analysis of variance was conducted following the statistical model: Y_ijk_ = m + B/A_jk_ + A_j_ + G_i_ + GA_ij_ + e_ijk_, in which Y_ijk_ is the observation of the i-th genotype in the j-th block in the k-th block; m is the general constant; B/A_jk_ is the effect of the k-th block in the j-th environment; A_j_ is the fixed effect of the j-th environment (P level); G_i_ is the fixed effect of the i-th genotype; GA_ij_ is the fixed effect of the interaction between the i-th genotype and the j-th environment; e_ijk_ is the experimental error associated with the observation Y_ijk_ with NID (0, *σ*^2^).

Based on the averages of the 90 testcross hybrids, a diallel analysis was carried out in line with the methodology suggested by Griffing [56], adapted to partial diallels, as follows: Y_i j_ = m + g_i_ + g_j_ + s_ij_ + a_k_ + ga_ik_ + ga_jk_ + sa_ij_ + e_(k)ij_, in which Y_ij_: The average value of the hybrid combination, testers (g_i_), and lines (g_j_); m: The overall average of the hybrid combinations; g_i_: The general combining ability (GCA) effect of group i (progenies); g_j_: The GCA effect of group j (testers); s_ij_: The specific combining ability (SCA) effect for the crosses between parents of the orders i and j; a_k_: The effect of the environments k; ga_ik_ and ga_jk_: The interaction effects between the GCA associated with the i-th and j-th parents in the environments k; sa_ij_: The interaction effect of SCA associated with parents i and j and environments k; and e_(k)ij_: The average experimental error.

The statistical analyses were conducted using the software GENES (v.1, Universidade Federal de Viçosa, MG, Brazil) [57].

## 5. Conclusions

Phosphorus use efficiency is mostly dependent on additive gene effects in the expression of popping expansion, and conversely, dominance gene effects in the expression of GY and PV.

The best strategy to obtain efficient and responsive genotypes in phosphorus use involves the exploration of heterosis, with parents that provide an accumulation of additive genes for popping expansion.

Lines L688 and L689 and testers P7 and L59 showed additive gene effects and are highly recommended for use in future hybrid combinations looking to increase GY and PE in environments with low soil-phosphorus levels.

The genetic merit of the hybrids enabled us to know the combinations with the highest dominance gene effects for efficiency and responsiveness in phosphorus use for the traits GY, PE, and PV, as follows: L688 × L70, L694 × L80, L688 × L70; L694 × L80, L689 × L59, L694 × L80; L689 × P7, and L689 × L59, in that order.

Due to the good performance of the parents originating from the UENF-14 population, L688, L694, and L689 are selected as the standards for forming a new heterotic group and for “line recycle”, creating new biparental populations.

The production of a triple hybrid by crossing the simple hybrid L688 × L689—from the UENF-14 heterotic group—with the L80 line—from the Viçosa: UFV genealogy—is a good alternative for meeting the needs of the North and Northwest regions of Rio de Janeiro State and for achieving sustainable agriculture.

## Figures and Tables

**Table 1:** Summary of the analysis of variance, of the general combining ability (GCA), and of the specific combining ability (SCA) for the agronomic traits evaluated in testcrosses under contrasting conditions of phosphorus in soil in Campos dos Goytacazes and Itaocara.

SV	DF	Mean Squares					
		Campos dos Goytacazes			Itaocara		
		GY	PE	PV	GY	PE	PV
**Block/Phosphorus**	4	554,624.5	9.5	529.0	103,022.6	3.3	116.5
Genotype (G)	89	954,080.6 **	69.6 **	1101.1 **	992,731.8 **	87.40 **	1414.1 **
GCA I	14	1,672,189.3 **	247.9 **	2585.7 **	2,244,297.7 *	314.3 **	4149.6 **
GCA II	5	4,395,517.2 *	279.9 **	2618.4 ^ns^	4,047,292.2 *	398.7 **	5616.3 *
SCA	70	564,640.9 **	18.9 **	695.8 **	524,233.5 *	19.8 ^ns^	566.9 ^ns^
Phosphorus (P)	1	36,456,840.8 **	246.3 **	37,105.2 **	17,515,196.9 **	255.7 **	27,642.8 **
G × P	89	330,925.0 **	14.6 **	317.0 **	423,063.8 **	16.7 **	509.3 **
GCA I × P	14	439,193.5 **	33.0 **	287.6 ^ns^	423,063.9 **	13.9 **	848.3 **
GCA II × P	5	684,529.0 **	20.6 *	956.3 **	845,321.8 **	8.8 **	868.8 **
SCA × P	70	284,013.2 **	10.5 **	277.2 **	727,343.1 **	17.8 **	415.9 **
Residue	356	178,327.5	7.0	179.3	209,610.6	2.8	171.1
**Mean**		2514.8	27.6	69.6	3143.7	26.2	82.4
**CVe (%)**		16.8	9.6	19.2	14.6	6.4	15.9

** and *—significant at 1% and 5% level probability using the F test; and ^ns^—not significant at 5% probability using the F test; SV—source of variation; DF—degree of freedom; CVe (%)—experimental coefficient of variation; PE—popping expansion; GY—grain yield; and PV—expanded popcorn volume per hectare.

**Table 2:** Description of the 21 popcorn genotypes from the Active Germplasm Bank of UENF used in the experiments in the North and Northwest regions of Rio de Janeiro State, Brazil.

Tester		Genealogy			Climate Adaptation			Classification Regarding P Use			
**L59**		**Beija-Flor: UFV**			**Temperate/Tropical**			**Efficient and Responsive**			
**L70**		**BRS Angela: EMBRAPA**			**Tropical**			**Efficient and Responsive**			
P7		Híbrido Zaeli			Temperate/tropical			Efficient and responsive			
L54		Beija-flor: UFV			Temperate/tropical			Inefficient and non-responsive			
L75		Viçosa: UFV			Temperate/tropical			Inefficient and non-responsive			
L80		Viçosa: UFV			Temperate/tropical			Inefficient and non-responsive			
**IRS Progenies**		**Genealogy and** **Climate Adaptation**		**IRS Progenies**		**Genealogy and** **Climate Adaptation**		**IRS Progenies**		**Genealogy and** **Climate Adaptation**	
1	L681	UENF-14	Tropical	6	L686	UENF-14	Tropical	11	L692	UENF-14	Tropical
2	L682	UENF-14	Tropical	7	L688	UENF-14	Tropical	12	L693	UENF-14	Tropical
3	L683	UENF-14	Tropical	8	L689	UENF-14	Tropical	13	L694	UENF-14	Tropical
4	L684	UENF-14	Tropical	9	L690	UENF-14	Tropical	14	L695	UENF-14	Tropical
5	L685	UENF-14	Tropical	10	L691	UENF-14	Tropical	15	L696	UENF-14	Tropical

UFV—Universidade Federal de Viçosa; UENF—Universidade Estadual do Norte Fluminense Darcy Ribeiro; EMBRAPA—Brazilian Agricultural Research Corporation; and IRS—Intrapopulation Recurrent Selection.

**Table 3:** Chemical and particle-size analysis of the soil at 0–10 and 10–20 cm depths in Campos dos Goytacazes and Itaocara.

Local	Soil Layer	pH	P	K	Ca	Mg	Al	Na	C	OM	CEC	BS	V	Clay
		H_2_O	mg/dm^3^	mmol_c_/dm^3^					g/dm^3^		mmol_c_/dm^3^		%	g/dm^3^
**Campos dos** **Goytacazes**	0–10 cm	5.7	4.0	3.4	14.6	8.3	0.0	1.3	7.6	13.1	48.9	27.6	56.0	305.0
	10–20 cm	5.4	3.0	2.3	14.4	6.7	0.9	1.2	7.2	12.4	44.5	24.6	55.0	
**Itaocara**	0–10 cm	5.2	5.0	1.2	38.9	30.0	1.2	0.6	14.9	25.7	103.8	70.7	68.0	140.0
	10–20 cm	5.3	3.0	0.7	36.2	31.0	1.4	0.4	11.0	19.0	96.2	68.3	71.0	

## Data Availability

Not applicable.
